# Lipase catalyzed epoxidation of fatty acid methyl esters derived from unsaturated vegetable oils in absence of carboxylic acid

**DOI:** 10.1186/s13065-018-0409-2

**Published:** 2018-04-11

**Authors:** Alejandro Sustaita-Rodríguez, Víctor H. Ramos-Sánchez, Alejandro A. Camacho-Dávila, Gerardo Zaragoza-Galán, José C. Espinoza-Hicks, David Chávez-Flores

**Affiliations:** Facultad de Ciencias Químicas, Universidad Autónoma de Chihuahua, Nuevo Circuito Universitario, 31125 Chihuahua, Mexico

**Keywords:** Epoxidation, FAME, Lauric acid, Lipase

## Abstract

Nowadays the industrial chemistry reactions rely on green technologies. Enzymes as lipases are increasing its use in diverse chemical processes. Epoxidized fatty acid methyl esters obtained from transesterification of vegetable oils have recently found applications as polymer plasticizer, agrochemical, cosmetics, pharmaceuticals and food additives. In this research article, grapeseed, avocado and olive oils naturally containing high percents of mono and poly unsaturations were used as starting materials for the production of unsaturated fatty acid methyl esters. The effect of lauric acid as an active oxygen carrier was studied on epoxidation reactions where unsaturated fatty acid methyl esters were converted to epoxy fatty acid methyl esters using immobilized *Candida antarctica* Lipase type B as catalyst and hydrogen peroxide as oxygen donor at mild temperature and pressure conditions. After this study it was confirmed by ^1^H NMR, ^13^C NMR and GC–MS that the addition of lauric acid to the enzymatic reaction is unnecessary to transform the alkenes in to epoxides. It was found that quantitative conversions were possible in despite of a carboxylic acid absence.

**Electronic supplementary material:**

The online version of this article (10.1186/s13065-018-0409-2) contains supplementary material, which is available to authorized users.

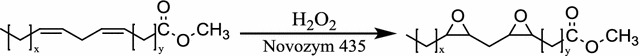

## Introduction

In response to a serious concern over the depleting fossil reserves and the negative environmental impact caused by their products and processes, there has been a growing trend towards utilization of renewable feedstocks as a source of chemicals and materials [1]. Vegetable oils and the product of their transformations (fatty acids and alkyl esters) are important raw materials for producing a variety of products such coatings, paints, lubricants, soaps [2], and inks [3]. For more complex molecules such polymers, copolymers and their composites, the oils have to be chemically modified. One of the most interesting functional group for this purpose is the epoxy ring. Epoxides are major key raw materials for various industrial products such as polymer plasticizer, agrochemical, cosmetics, pharmaceuticals and food additives. Epoxidation consists on the formation of an oxirane (epoxy) group by the reaction of peroxyacids (peracids) and olefinic double bonds [4]. Epoxides derived from vegetable oils are used as PVC stabilizers, plasticizers and in polyurethane production. They can also be useful as reactive diluents for paints, production of surfactants, corrosion protections agents and additives to lubricants. Using epoxidized FAMEs from unsaturated vegetable oils it is possible to obtain polymers and composites with better mechanical, electric, thermal properties than those of the polymers obtained from petrochemical products and greater resistance to oxidation than the latter ones [5–7].

During industrial epoxidation, the required peroxyacids are commonly produced by the reaction of acetic or formic acids and hydrogen peroxide in presence of strong mineral acids, such as H_2_SO_4_ and H_3_PO_4_ or by the direct addition of any peroxyacid which can cause equipment corrosion and promote undesirable oxirane ring-opening reactions [8, 9].

Recently, enzymatic epoxidation reactions have been better accepted compared with chemical synthesis due to the milder reactions conditions as formation of stable hydroperoxides directly from fatty acids, high stereoselectivity, significant suppression of side reactions and high conversion [10–12].

## Materials and methods

Lipase B from *Candida antarctica* (Novozym 435) immobilized on macroporous polyacrylate resin beds, chloroform-*d*, cyclohexane, isooctane, dodecanoic acid (lauric acid) and pentadecanoic acid were acquire from Sigma-Aldrich. Potassium hydroxide, hydrogen peroxide 30% was purchased from J.T. Baker, toluene was acquired from Fermont, sodium hydroxide, potassium sulphate and anhydrous ethanol and methanol 99% were purchased Mallinckrodt. Vegetable oils (grapeseed, avocado and olive oils) were gotten from a local market (Additional file 1).

### Synthesis procedure

All experiments on this report were made at least by triplicate. Synthesis of epoxides involved two steps: in the first stride, different vegetable oils (grapeseed, avocado and olive) were converted into methyl esters by transesterification reaction using KOH as catalyst and methanol as acyl acceptor.

In a second step 1 g of methyl esters, 100 mg of Novozym 435, 1 mmol of lauric acid and 6 mL of toluene were added into a 50 mL round bottom flask, and then 5 mL of distillate water was added to the reaction mixture to create a biphasic system which was heated to 45 °C and homogenized with stirring. Once the temperature was reached and kept for 5 min, slowly 1 mL of hydrogen peroxide was added to the reaction system and the reactions were stirred at this temperature for 16 h. Upon completion of reaction time, samples were cooled to 4 °C and Novozym 435 was recovery by vacuum filtration, using filter paper number 41. The filtered reaction mixture was firstly washed several times with distilled water and the separated organic layer was dried with anhydrous sodium sulphate for 1 h to remove trace water and decompose traces of unreacted peroxides. Finally, product was separated from solvent using a vacuum rotary evaporator at 90 °C for 1 h. Experiments were repeated in absence of lauric acid under the same conditions of reactions.

### Characterization of FAMEs and its epoxides

Fatty acid methyl esters (FAMEs) and its epoxidized products were analysed by gas chromatography (Agilent series 7890B) coupled with mass spectrometer (Agilent series 5975C) in a HP-INNOWAX column, 30 m × 0.25 mm, 0.25 μm and by nuclear magnetic resonance trough ^1^H-NMR and ^13^C-NMR experiments using a Bruker 400 MHz magnet and deuterated chloroform solvent for sample preparation.

## Results and discussion

### FAMEs characterization

**Fig. 1:**
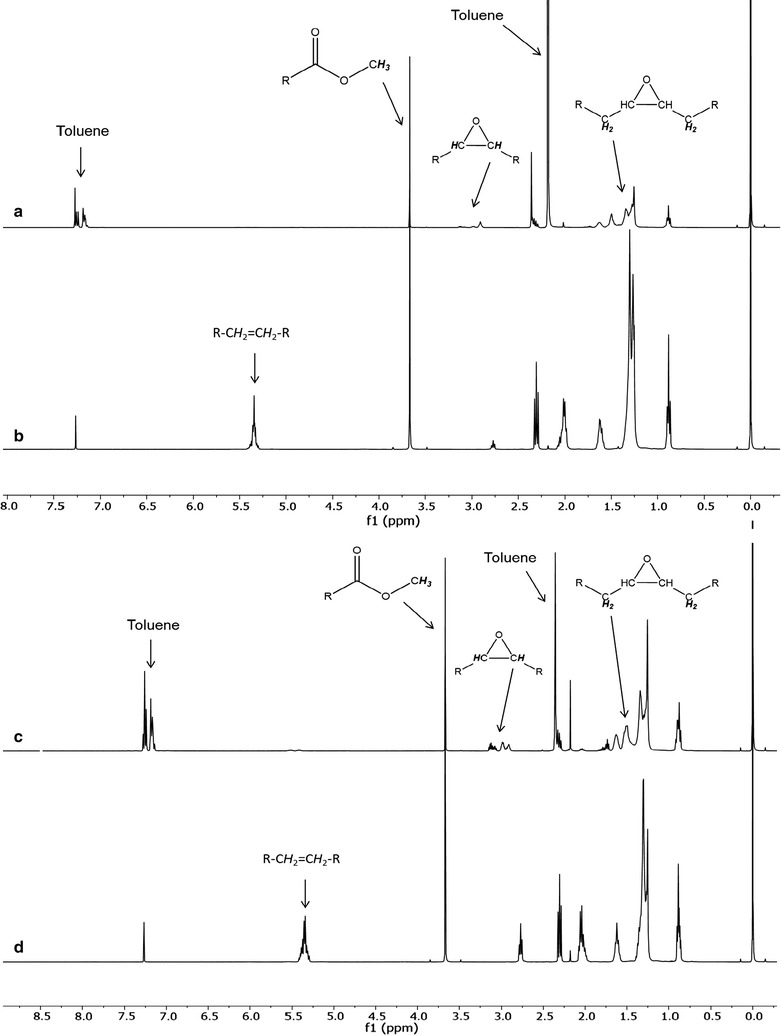
^1^H-NMR spectrum of **a** avocado epoxide, **b** FAME avocado, **c** grapeseed epoxide, **d** FAME grapeseed

**Fig. 2:**
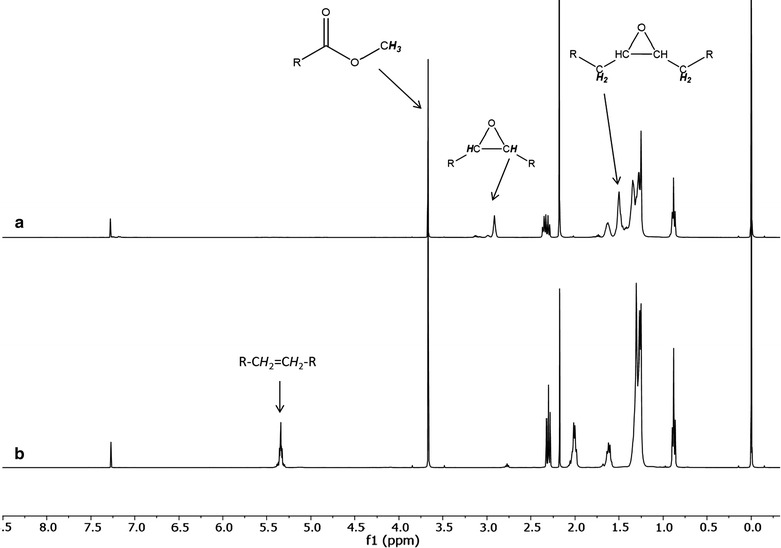
^1^H-NMR spectrum of **a** olive epoxide and **b** FAME olive

FAMEs from different vegetable oils were characterized by GC–MS, ^1^H-NMR and ^13^C-NMR analytical techniques. A proton NMR spectrum is shown on Figs. 1a, c, d and 2, in which can be observed the characteristic peak of methoxy protons as a singlet at 3.67 ppm. Also, is possible observe a triplet at 2.30 ppm that indicates presence of carbonyl α-CH_2_ protons. These two peaks confirmed the presence of methyl esters and its integration areas confirms the conversion on each sample. Other peaks were observed at 0.88 ppm for terminal or deshielded methyl protons, 1.27 ppm for methylene protons ay the alkyl chain and a signal at 1.62 ppm was observed owing to β-carbonyl methylene protons. Protons on the insaturation or double bonds were identified at 5.34 ppm.

FAMEs conversion (Table 1) was determined choosing relevant signals for integration (methoxy protons at 3.67 ppm and α-CH_2_ protons at 2.30 ppm). The equation used to quantify the conversion was [13]:

1 $${\text{C}} = \frac{{2A_{Me} }}{{3A_{{{\text{CH}}_{2} }} }}(100\% )$$

where: C = percentage conversion of triglycerides to the corresponding methyl esters; A_Me_ = integration value of the methoxy protons of the methyl esters; ${\text{A}}_{{{\text{CH}}_{2} }}$ = integration value of α-methylene protons.

**Table 1:** Conversion and percentage of saturated, mono and polyunsaturated FAMEs derived from vegetable oils

Oil	C (%)	SFAME (%)	MUFAME (%)	PUFAME (%)
Olive	95.19	13.46	78.84	7.69
Avocado	97.43	8.17	77.40	14.42
Grapeseed	96.74	14.67	19.92	65.41

Saturated fatty acid methyl ester (SFAME), monounsaturated fatty acid methyl ester (MUFAME) and polyunsaturated fatty acid methyl ester (PUFAME) content (Table 1) was calculated using the integral for different protons [14] and the equations are given below:

2 $${\text{PUFAME}} = \left( {{\text{Z}}/{\text{Y}}} \right)$$

3 $${\text{MUFAME}} = \left[ {\left( {{\text{X}}/ 2 {\text{Y}}} \right) - {\text{PUFAME}}} \right]$$

4 $${\text{SFAME}} = \, \left[ { 1- \left( {{\text{X}}/ 2 {\text{Y}}} \right)} \right]$$

### Effect of lauric acid in enzymatic epoxidation of FAMEs from vegetable oils

Initial experiments were performed in presence of carboxylic acid as lauric acid because has been reported that the use of linear chain fatty acids favours the reaction conversion into the corresponding epoxide due to the fatty acids acts as precursor in the epoxide formation [15–17]. Originally FAMEs from different vegetable oils did not contain epoxy groups but after epoxidation reactions their presence could be identified with chemical shifts due to double bonds (~ 5 ppm). Figures 1b, d and 2b shows the disappearance of this peaks which means epoxidation was completely carried out (conversion > 99%). A new signals at 2.90 ppm confirmed the existence of an epoxy ring corresponding to methine CH proton and at 1.50 ppm corresponding to the new methylene group alpha to the new epoxy group. Finally, signal at 1.63 ppm indicates a methylene group between the two new epoxy groups.

**Fig. 3:**
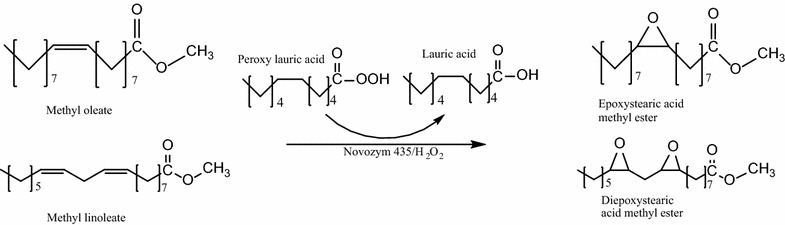
Reaction mechanism of epoxystearic and diepoxystearic acid methyl ester from methyl oleate and methyl linoleate

In this case, the use of lauric acid in the reaction system and its effect can be explained according to the proposed reaction mechanism shown in Fig. 3 (methyl oleate is used as example because of is the mainly component in FAMEs derived from olive and avocado oils while methyl linoleate corresponds to FAME derived from grapeseed oil) in which the enzyme catalyzed the formation of peracid from lauric acid and H_2_O_2_, then perlauric acid transfers the oxygen to the double bond in FAMEs to yield epoxystearic and diepoxystearic acid methyl ester respectively.

Experiments were also performed in absence of lauric acid in order to evaluate its effect in the epoxide formation. For these assays characteristics signals on the NMR spectra corresponding to epoxides were identified at 2.90, 1.50, and 1.63 ppm as in the previous experiments. Also, it was noticed the disappearance of peak belonging to double bonds (~ 5 ppm) where it can be inferred that conversion was more than 99% such as in the previous experiments. All ^1^H NMR signals from FAME and epoxides of FAME matched perfectly with previous reports [18].

**Fig. 4:**
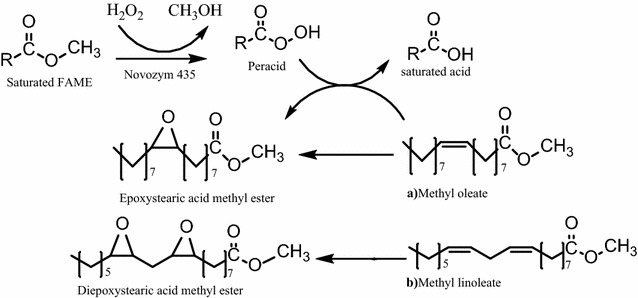
Reaction mechanism of FAMEs epoxidation in absence of lauric acid

Several researches have reported that addition of fatty acids in reaction system is crucial to carry out successful epoxidations but according to the results obtained from this paper it is possible stablish that lauric acid or any fatty acid is not critical or crucial to get high epoxidation or conversions. As a likely explanation, in Fig. 4 is shown the proposed reaction mechanism to describe the pathway in which enzymatic epoxidation without lauric acid is accomplished.

Reaction mechanism involves two steps: Firstly, saturated and insaturated methyl esters present in the mixture of FAMEs from vegetable oils react with H_2_O_2_ to form the corresponding peracid, secondly peracid spontaneously donates the oxygen to a double bond of the unsaturated FAMEs (methyl oleate and methyl linoleate are used as example) to yield epoxystearic and diepoxystearic acid methyl ester respectively.

**Fig. 5:**
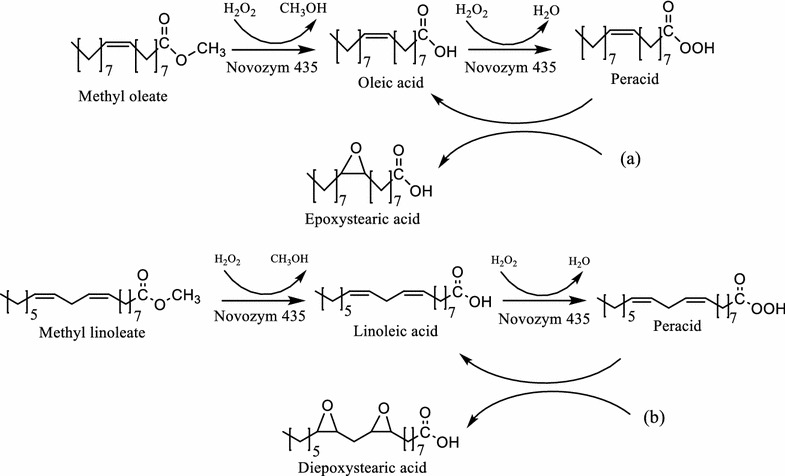
Reaction mechanism of FAMEs hydrolysis to yield epoxides

As alternative to the mechanism given in Fig. 4, it is proposed a hydrolysis of the ester bond as a side reaction (Fig. 5). In this case Novozym 435 catalyses the hydrolysis of ester to produce its corresponding acid which is oxidized by the enzyme itself to form a peracid. Finally, peracid donates the oxygen to double bond and epoxides are brought about. Therefore, the final product will be a mixture of epoxidized methyl esters and fatty acids [1].

GC/MS analysis was used to evaluate the resulting epoxides structures on proposed mechanism. The epoxy stearate (9,10-epoxy octadecanoate) was identified at RT: 33.69 min and the mass spectrum allowed to define its molecular structure. The molecular ion (M^+^) was detectable at 312, the major fragments m/z 155 (M-157; loss of ·(CH_2_)_7_-COOCH_3_) and 199 (M-113; loss of ·(CH_2_)_7_-CH_3_) represent the result of cleavage at the epoxy ring [19].

The corresponding peak to diepoxy stearate (9,10-12,13-diepoxy octadecanoate) appeared at RT: 37.38 min and the molecular ion (M^+^) was perceptible at 326. The spectrum was similar at MS spectrum previous reported for the same compound [18, 20]. Characteristic peaks were identified at m/z 295 (M-31), 277 (M-49), 255 [CH_2_CH(O)CHCH_2_CH(O)CH(CH_2_)_7_COOCH_3_-14]^+^, 237 [CH_2_CH(O)CHCH_2_CH(O)CH(CH_2_)_7_COOCH_3_-32]^+^, and 211[O=CHCH_2_CH(O)CH(CH_2_)_7_COOCH_3_-31]^+^.

## Conclusion

It is possible to obtain epoxides fatty acid methyl esters by an enzymatic reaction under mild temperature and pressure conditions (45 °C, 1 atm., 250 rpm) and high selectivity by the use of Novozym 435 as biocatalyst and hydrogen peroxide as oxygen donor. The reactions were carried out with conversions higher than 99%. This study also demonstrates that the addition of a carboxylic acid is not essential or crucial to perform epoxidation reactions successfully due to the fact that the unsaturated FAMEs present in the mixture generated by enzymatic hydrolysis act as precursors in the epoxide formation.

## Additional file

**Additional file 1.** Additional figures.

## Abbreviations

FAMEs: fatty acid methyl esters
PVC: polyvinylchloride
SFAME: saturated fatty acid methyl ester
MUFAME: monounsaturated fatty acid methyl ester
PUFAME: polyunsaturated fatty acid methyl ester
