# Supplementary material for: Lipase catalyzed epoxidation of fatty acid methyl esters derived from unsaturated vegetable oils in absence of carboxylic acid
Source: Chem Cent J. 2018 Apr 11;12:39. doi: 10.1186/s13065-018-0409-2 (PMC5895561; doi:10.1186/s13065-018-0409-2)
Supplement: Supplementary file 1 — Additional file 1. Additional figures. [file 13065_2018_409_MOESM1_ESM.docx]

**Additional file JCC**

**MS Spectrums**

**(M-113)**

**(M-157)**

**Figure S1. Mass spectrum of epoxy stearate (9,10-epoxy octadecenoate).**

**237**

**M^+^=326**

**237**

**211** (*O=CHCH_2_CH(O)CH(CH_2_)_7_COOCH_3_-31)*

**277(M-49)**

**295(M-31)**

**255**

**Figure S2. Mass spectrum of diepoxy stearate (9,10-12,13-diepoxy octadecenoate).**

**Figure S3. Chromatogram in presence of lauric acid that indicates epoxide formation in RT at 33.69min derived from avocado oil.**

**Figure S4. Shows the chromatogram that indicates epoxide formation derived from avocado oil in absence of lauric acid. RT 33.70**

**Figure S5. Chromatogram of epoxide formation derived from grape oil in presence of lauric acid. RT at 34.43 indicates monoepoxystearate and RT at 39.41 indicates formation of diepoxystearate.**

**Figure S6. Chromatogram of epoxide formation in absence of lauric acid from grape oil.**

**Figure S7. Chromatogram of epoxide formation in presence of lauric acid derived from olive oil.**

**Figure S8. Chromatogram of epoxide formation in absence of lauric acid derived from olive oil.**

**Epoxidized products were analysed by gas chromatography (series 7890B) coupled with mass spectrometer (series 5975C) in capillary column VF-5ms, 30m x 0.25mm, 1μm And by ^1^H-NMR.**

**The method was 120°C during 2 min, 5°C/min to 200°C, 5°C/min to 270°C and hold to 270 for 10min.**
